# Proliferative diabetic retinopathy in acromegaly

**DOI:** 10.4103/0974-620X.64237

**Published:** 2010

**Authors:** Chintan Malhotra

**Affiliations:** Department of Ophthalmology, Himalayan Institute of Medical Sciences, Dehradun (Uttrakhand), India

**Figure 1:**
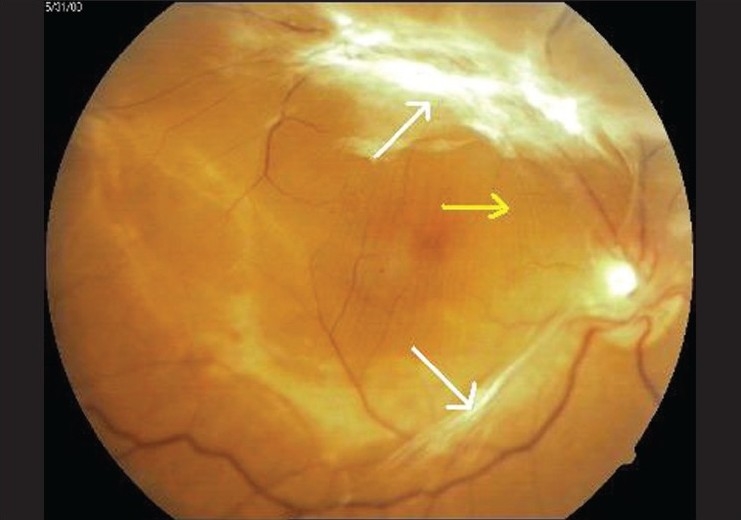
Fundus photograph right eye - fibrovascular traction bands extending from optic disc, along both superior and inferior temporal arcades (white arrows) with taut posterior hyaloid membrane (yellow arrow) leading to folds in internal limiting membrane

**Figure 2:**
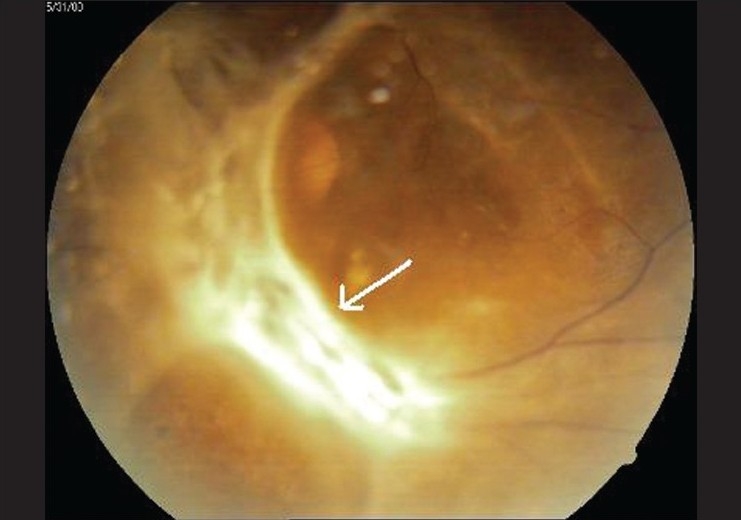
Fundus photograph left eye - extensive gliosis covering optic disc (white arrow) and extending along the superior and inferior vascular arcades

A 55-year-old male presented to the outpatient services of the Ophthalmology department with complaints of gradual, painless progressive diminution of vision both eyes (OU) for the last two years. Ocular examination showed best corrected visual acuity to be counting fingers at a distance of two metres OU. Anterior segment findings were unremarkable OU. Examination of the fundus revealed bilateral severe proliferative diabetic retinopathy with active fibrovascular membranes extending from the optic disc along both superior and inferior temporal arcades and also nasal to the optic disc [Figures 1 and 2]. Taut posterior hyaloid membrane was present OU. The patient was unaware of his diabetic status and had no family history of diabetes. However he reported noticing coarsening of his facial features, deepening of the voice and enlargement of hands and feet for the last two to three years.

**Figure 3:**
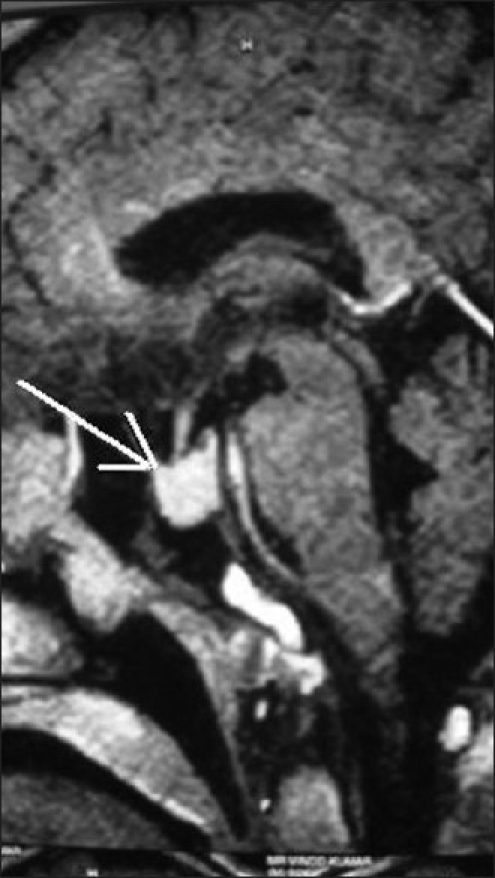
Contrast enhanced MRI scan (saggital section) - intrasellar pituitary macroadenoma (white arrow) involving the anterior lobe of the pituitary with no parasellar or suprasellar extension

On systemic examination by an internist the patient had multiple clinical features of acromegaly including enlarged hands and feet, mandibular enlargement, prognathism, coarse facial features, large fleshy nose, deep and hollow sounding voice, arthropathy, skin tags, acanthosis nigricans and weakness of proximal muscles. X-ray sella (cone down view) showed widening of the sella with destruction of the floor. A magnetic resonance scan revealed the presense of an intrasellar pituitary macroadenoma (1.4 × 1.1 cm) involving the anterior lobe of the pituitary with no parasellar or suprasellar extension [Figure 3].

Blood pressure was recorded to be 150/100mm Hg. Biochemical investigations revealed fasting blood sugar level varying between260-320mg/ml, glycosylated Hb (Hb A1c) > 8%., serum GH 42 ng/ml, free T3 2.9 pg/ml, free T4 1.1 ng/ml, TSH 3.7 microIU/ml. The patient was diagnosed to have intrasellar pituitary macroadenoma with acromegaly (clinically active disease) associated with secondary hypertension and diabetes mellitus and severe proliferative diabetic retinopathy. Trans sphenoidal resection of the pitutary adenoma was advised to be followed by vitrectomy for management of ocular complications.

Acromegaly leads to development of secondary diabetes mellitus in 25-55.9% of cases.[1] The hyper secretion of growth hormone (GH) exposes peripheral organs such as the retina and kidney to conditions favoring the expression of growth-hormone-dependent growth factors such as insulin growth factor I (IGF-I) which may contribute to the development of diabetic microvascular disease by autocrine and/or paracrine effects[1] However reported incidence of diabetic retinopathy in acromegaly is low[2] and severe retinopathy is uncommon.[3]

Elevated levels of IGF-I in the vitreous cavity (5-8 ng/ml) and serum (80-220 ng/ml) have been demonstrated in patients having proliferative diabetic retinopathy associated with primary diabetes.[4] Inokuchi and associates have reported much higher levels of vitreous IGF-I (14 ng/ml) and serum IGF-I (726 ng/ml) in a case of acromegaly with severe proliferative diabetic retinopathy.[5] They suggest that the excessive production of local IGF-I or the breakdown of the blood-ocular barrier (which results in the diffusion of serum IGF-I into the vitreous cavity), may occur in such patients which accelerates the progression of the retinopathy. Other investigators have reported that IGF-I accelerates the expression of VEGF in some cell lines.[6] Smith and associates have reported that inhibition of GH can inhibit ischaemia induced retinal neovascularisation *in vivo* via vascular endothelial growth factor (VEGF).[7] Thus growth hormone may play a major role in the progression of diabetic retinopathy in combination with IGF-I and VEGF.

In our case there was severe proliferative diabetic retinopathy associated with acromegaly. Patients with acromegaly should therefore be kept under close observation to detect the occurrence of diabetic retinopathy which though uncommon may be of the proliferative type.
